# Risk of Melanoma in People with HIV/AIDS in the Pre- and Post-HAART Eras: A Systematic Review and Meta-Analysis of Cohort Studies

**DOI:** 10.1371/journal.pone.0095096

**Published:** 2014-04-16

**Authors:** Catherine M. Olsen, Lani L. Knight, Adèle C. Green

**Affiliations:** 1 Department of Population Health, QIMR Berghofer Medical Research Institute, Brisbane, Queensland, Australia; 2 Institute of Inflammation and Repair, Manchester Academic Health Sciences Centre, University of Manchester, Manchester, United Kingdom; King's College London, United Kingdom

## Abstract

**Objective:**

Following the introduction of highly active antiretroviral therapy (HAART) the risk of AIDS-defining cancers decreased but incidence of many non-AIDS-defining cancers has reportedly increased in those with HIV/AIDS. Whether melanoma risk has also changed in HIV/AIDS patients post-HAART is unknown and therefore we evaluated this in comparison with the risk before HAART.

**Design:**

Systematic review and meta-analysis.

**Methods:**

We searched Medline, Embase and ISI science citation index databases to April 2013. All cohort studies of patients diagnosed with HIV/AIDS that permitted quantitative assessment of the association with melanoma were eligible. Detailed quality assessment of eligible studies was conducted, focussing particularly on adjustment for ethnicity, a priori considered essential for an unbiased assessment of melanoma risk. Data were pooled using a random effects model.

**Results:**

From 288 articles, we identified 21 that met the inclusion criteria, 13 presenting data for the post-HAART era and 8 for the pre-HAART era. Post-HAART the pooled relative risk (pRR) for the association between HIV/AIDS and melanoma was 1.26 (95% CI, 0.97–1.64) and 1.50 (95% CI 1.12–2.01) among studies that accounted for ethnicity, with evidence of significant heterogeneity (P = 0.004, I^2^ = 55.5). Pre-HAART pRRs were 1.26 (95% CI 1.11–1.43; P_het_ = 0.82) and 1.28 (95% CI 1.10–1.49) among studies adjusted for ethnicity.

**Conclusions:**

People with HIV/AIDS remain at a significantly increased risk of developing melanoma in the post-HAART era. White skinned people with HIV/AIDS should be screened regularly and counselled against excessive sun exposure.

## Introduction

Prior to the introduction of highly active antiretroviral therapy (HAART) in the latter part of the 1990s, a modestly increased incidence of cutaneous melanoma in people with human immunodeficiency virus infection/acquired immunodeficiency syndrome (HIV/AIDS) was observed. A meta-analysis of population-based data collected mostly prior to the introduction of HAART reported an overall Standardised Incidence Ratio (SIR) for melanoma of 1.24 (95% CI = 1.04-1.48) [1]. Since then there has been a decrease in incidence of AIDS-defining cancers including Kaposi's sarcoma and non-Hodgkin lymphoma, but a reported increase in incidence of many non-AIDS-defining cancers [2]. Whether the risk of melanoma in HIV/AIDS populations compared with the general population has increased since the introduction of HAART along with other non-AIDS-defining cancers is unknown however. It is important to quantify this risk given increased longevity of patients treated with HAART [3] and the consequent increased period at risk of melanoma against a background of rising rates in most white populations [4] together with some evidence of patients' predilection for recreational sun exposure [5]. The association between HIV and melanoma is also important to understand better, since laboratory studies have indicated a role for immune surveillance in melanoma genesis and increasingly immunotherapeutic approaches for the treatment of metastatic disease are being developed [6]. We conducted a systematic review and meta-analysis to evaluate melanoma risk in HIV/AIDS patients compared with the general population in the pre-HAART and post-HAART eras.

## Patients and Methods

The systematic review and meta-analysis was conducted in accordance with the Meta-analysis of Observational Studies in Epidemiology guidelines for reviews of observational. Studies [7], and we followed the PRISMA statement [8] to guide reporting.

### Data Sources and Searches

Eligible studies up to April 2013 were identified by searching the Medline 1950 (U.S. National Library of Medicine, Bethesda, MD) database using PubMed software as the search interface; Embase 1966 database (Elsevier Science, Amsterdam, Holland) using the Embase search interface; and the ISI Science Citation Index using the ISI Web of Science search interface. We used the following medical subject headings terms or text words (both the United States and United Kingdom spellings): melanoma, cancer, neoplasms, HIV, AIDS, human immunodeficiency virus, acquired immunodeficiency syndrome, aetiology, cohort (Appendix S1 – Search terms). Studies that had been commonly cited in the literature and review articles were also included as citation search terms in the ISI Science Citation Index (1990 to present) to identify subsequent studies that had referenced them. The search was not limited to studies published in English. We read the abstracts of all identified studies to exclude those that were clearly not relevant. The full texts of the remaining articles were read to determine if they met study inclusion criteria. Eligible studies were also identified by hand-searching the reference lists of retrieved articles.

### Study Selection

We included cohort studies, including population-based record linkage studies, of adult populations (ie predominantly > 18 years of age) diagnosed with HIV/AIDS that permitted quantitative assessment of the association between HIV/AIDS and melanoma. Studies that reported different measures of relative risk (RR), namely Hazard Ratio (HR), Incidence Rate Ratio (IRR), and Standardised Incidence Ratio (SIR) were included. Excluded were studies reporting on paediatric cohorts (< 18 years of age) and studies with melanoma mortality as the outcome. Any discrepancies between investigators about inclusion of a study were resolved by joint evaluation of the manuscript. When multiple reports were published on the same population or subpopulation, we included the report with the longest follow-up duration or the largest population.

### Data Extraction and Quality Assessment

Two investigators (CO, LK) independently abstracted data from identified studies using a standardised data abstraction form, with inconsistencies resolved by consensus. The following information was recorded for each study: design, location, years of data collection, source and definition of cohort, number of cases, person-year duration of follow-up, age of study population, variables used for statistical adjustment, point estimates (RR, HR, IRR or SIR), and 95% confidence intervals (95% CI). Where several risk estimates were presented (e.g. crude and adjusted), we abstracted those adjusted for the greatest number of potential confounders. Studies that reported results separately by time period (pre- and post-HAART) or other strata with no combined data were treated as independent data sets in the meta-analysis.

Two investigators (CO, LK) independently evaluated the quality of the studies by using a scoring system that was designed with reference to the following guidelines: Meta-analysis Of Observational Studies in Epidemiology (MOOSE) [7], Quality Assessment Tool for Systematic reviews of Observational studies (QATSO) [9], and Strengthening the Reporting of Observational Studies in Epidemiology (STROBE) [10]. A total score of 4–6 was considered high quality, 1–3, low-moderate quality. One point each was allocated for (a) representativeness of the exposed cohort (i.e. population-based); (b) diagnosis of HIV confirmed according to accepted laboratory testing criteria [11]; (c) description of calculation of person-years at risk; (d) consistency of melanoma ascertainment in HIV cohort and comparison population; (e) adjustments made for age, sex and ethnicity; and (f) adjustments made for other relevant factors, namely time period, cancer registry and region. Single-institution studies and those with selected patient representation were classified as non- population-based. Disagreements about any item were resolved through discussion.

### Data Synthesis and Analysis

To pool individual study estimates for the risk of melanoma in HIV/AIDS in the pre-HAART and post-HAART time periods, we used random effects models [12]. The analyses were repeated using the weighted average method where the weight of each study is inversely proportional to the study variance (Table S1). Statistical heterogeneity among studies was assessed using the Q statistic [13] (significance level at P<0.05), and inconsistencies were quantified using the I^2^ statistic [14]. We performed a sensitivity analysis by omitting one study at a time, and calculated the pooled relative risk (pRR) for the remaining studies to evaluate whether the results were affected markedly by a single study. Finally, publication bias was evaluated through visual inspection of a funnel plot and with Begg's and Egger's tests [15], [16]. We defined the post-HAART was as after 1995. There was a rapid uptake of HAART in all countries in North America and Western and Central Europe [17] following the recommendations from an international panel on antiretroviral therapy for HIV infection, released in 1996 [18].

Subgroup analyses were carried out according to important study features: consistency of cancer ascertainment in cohort and comparison population; representativeness (population-based or clinic-based); quality score; geographic region; and adjustment for ethnicity. Since skin colour as indicated by ethnicity is strongly associated both with HIV/AIDS [19] and melanoma [20], ethnicity was considered a priori a major confounding factor and adjustment for ethnicity therefore regarded as essential for achieving an unbiased estimate of melanoma risk. A separate meta-analysis was conducted for studies that reported on risk of melanoma in cohorts of people diagnosed with AIDS where that population was clearly defined. All statistical analyses were performed using Stata Version 10 (Stata Corporation, College Station, TX).

## Results

### Search Results

**Figure 1:**
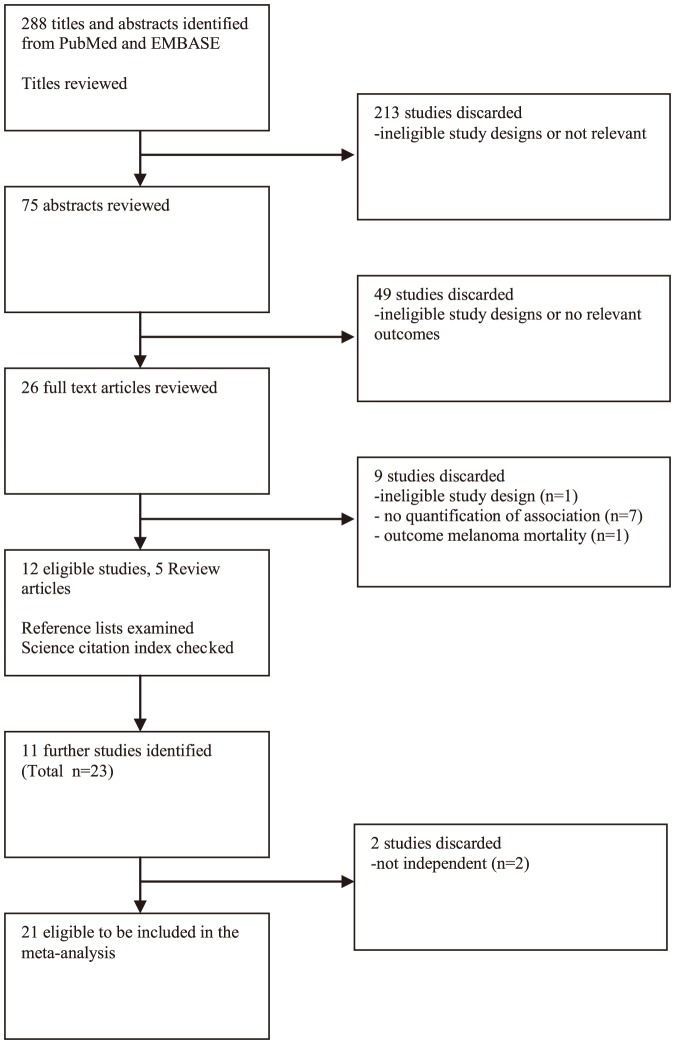
Flow chart of literature search for studies on the association between HIV/AIDS and risk of melanoma.

Details of the selection process for the eligible studies are shown in Figure 1. A total of 21 eligible studies were identified and included in our systematic review [2], [21]–[40]. We did not identify any relevant studies published in a language other than English, and no ineligible studies of children only were identified.

### Characteristics of included studies

Eligible studies were published between 1999 and 2013 (Table 1). One was conducted in Australia [32], eight in Europe [24], [26], [29], [31], [33]–[35], [39], and twelve in North America [2], [21]–[23], [25], [27], [28], [30], [36]–[38], [40]. Of the 21 studies, nine used different methods to ascertain melanoma diagnoses in the HIV cohort and the comparison population [2], [24], [25], [29], [30], [33], [35], [37], [39], the remaining 12 relied on cancer registry data for both groups [21]–[23], [26]–[28], [31], [32], [34], [36], [38], [40]. Most studies reported on cohorts of people either infected with HIV or diagnosed with AIDS. Six studies defined their cohort as patients with AIDS [22], [23], [27], [28], [31], [36], though four of these six studies included time before onset of AIDS in calculations of person-years at risk [22], [23], [28], [31]. The study by Frisch *et al.* [22] and one additional study [26] had conducted separate analyses on subsets of the population after AIDS onset. Five studies were not population-based: one cohort included only men who have sex with men [37], one included women only [25], one was a cohort of veterans [40] and two studies reported on single-clinic patient cohorts [2], [33]. The remaining 16 studies reported on population-based patient cohorts, predominantly men (range 76%–92%). Median follow-up time was reported by only eight [2], [24], [29], [30], [32], [37], [39] of the 21 included studies and ranged from two to ten years. Eight studies presented estimates for the pre-HAART [21]–[24], [27], [31], [32], [34] and 13 for the post-HAART time periods [2], [24], [27], [31]–[39], [41]. Findings of five studies that did not present effect estimates stratified by time period [25], [26], [28]–[30] were included in an overall estimate of risk based on all studies time period notwithstanding.

**Table 1:** Characteristics of the 21 studies included in the meta-analysis of HIV and/or AIDS and the risk of melanoma.

Study	Location	Study period	Cohort description	Cohort size	Cases	Male %	Cohort median age at recruitment	Age range	Effect estimates provided^b^		
									Pre-HAART	Post-HAART	AIDS
**Cohort studies**											
Calabresi *et al.*, 2013 [39]	Italy, 1 province	1999–2009	HIV or AIDS	5,090	8	82	35	19–64		SIR	
Vogel *et al.*, 2011 [35]	Germany, Bonn	1996–2009	HIV or AIDS	1,476	1	81	NS	16–85		SIR	
Seaberg *et al.*, 2010 [37]	United States, 4 metropolitan areas	1984–2007	HIV	6,949	9	100	32.6	<30–>50		SIR	
Franceschi *et al.*, 2010 [34]	Switzerland, 9 cancer registries	1985–2007	HIV or AIDS	9,429	11	81	32	>16	SIR	SIR	
Powles *et al.*, 2009 [33]	England, 1 clinical centre	1996–2007	HIV	11,112	5	90	NS	NS		SIR	
Long *et al.*, 2008 [2]	United States, 1 clinical centre	1996–2005	HIV	2,566	4	68	38	<30-> = 50		SIR	
Patel *et al.*, 2008 [30]	United States, 13 cancer registries	1992–2003	HIV	54,780	NS	76	NS	13–84			
Serraino *et al.*, 2007 [29]	Italy, 19 clinical centres France, 1 region	1988–2004	HIV	8074	2	81	31.3	<35->50			
Hessol *et al.*, 2004 [25]	United States	1994–1995	HIV or AIDS	1,554	2	0	NS	<30-50+			
Herida *et al.*, 2003 [24]	France, 12 cancer	1992–1999	HIV	77,025	19	84	Pre-HAART	15-69	SIR	SIR	
	registries						39 (male); 33 (female)				
							Post-HAART				
							42(male); 38 (female)				
**Record linkage studies**											
Silverberg *et al.*, 2011 [38]	United States, 1 state	1996–2008	HIV or AIDS	20,775	34	91	40.6	NS		RR	
Simard *et al.*, 2010 [36]	United States, 15 regions	1980–2004	AIDS	263,254	96	80	36	15-> = 50		SIR	SIR
Bedimo et al., 2009 [40]	United States	1997–2004	HIV or AIDS	33,420	96	98	NS	NS		IRR	
Dal Maso *et al.*, 2009 [31]	Italy, 24 cancer registries	1986–2005	HIV^a^ or AIDS	21,951	6		Pre-HAART 32	16–69	SIR	SIR	
							Post-HAART 38				
Van Leeuwen *et al.*, 2009 [32]	Australia, national	1982–2004	HIV or AIDS	17,175	53	92	NS	16–80	SIR	SIR	
Hessol *et al.*, 2007 [28]	United States, San Francisco	1990–2000	HIV^a^ or AIDS	14,210	36	96	NS	16–86			
Engels *et al.*, 2006 [27]	United States, 6 states and	1980–2002	AIDS	375,933	45	89	Pre-HAART 37.7	>15	SIR	SIR	SIR
	5 cities						Post-HAART 39.3				
Newnham *et al.*, 2005 [26]	England	1985–2001	HIV or AIDS	33,190 HIV 12,126 AIDS	2	79 HIV 88 AIDS	NS	15–60			SIR
Frisch *et al.*, 2001 [22]	United States, 11 regions	1978–1996	HIV^a^ or AIDS	302,834	145		37 (male) 35 (female)	15–69	SIR		SIR
Gallagher *et al.*, 2001 [23]	United States, New York State	1981–1994	HIV^a^ or AIDS	122,993	24	89	NS	15–69	SIR		
Cooksley *et al.*, 1999 [21]	United States, single county	1985–1994	HIV or AIDS	14,986	6	85	35	<1–91	SIR		

^a^HIV described if cohort entry was AIDS defined but person-year calculations included pre AIDS onset.

b Compared with the general population.

### Quality Assessment Results

Twelve (57%) of the 21 studies included in the meta-analysis were considered to be of high quality; the remaining 9 studies of low to moderate quality (Table S2). The most common limitation was lack of adjustment for ethnicity. Of the studies that had adjusted for ethnicity, Frisch *et al.* [22] provided detailed information on their method of adjustment but background cancer rates were not available for Hispanics and thus expected cancers in that ethnic group were based on cancer rates for whites (21% of both men and women in the cohort were Hispanic). Other studies reported that SIRs were adjusted for ethnicity [2], [23], [25], [27], [28], [36], [37], or matched [40], or a term for ethnicity was included in the regression model [30], [38].

### Outcome

**Figure 2:**
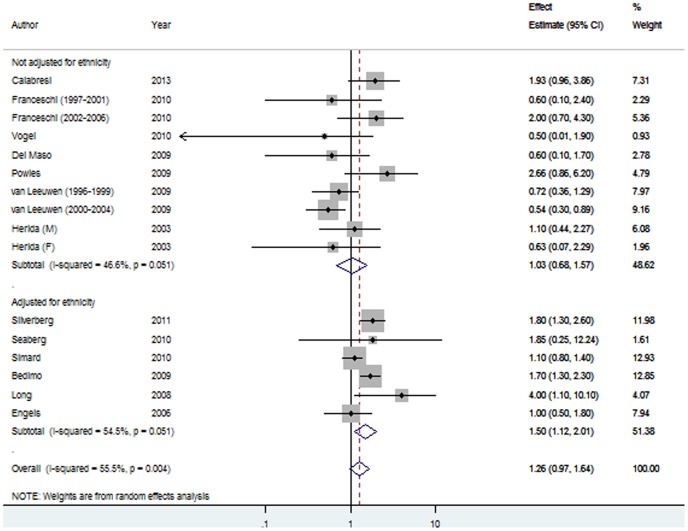
Forest plot of the association between HIV/AIDS and melanoma in the post-HAART time period, stratified by adjustment for ethnicity. Each line represents an individual study result with the width of the horizontal line indicating 95% CI, the position of the box representing the point estimate, and the size of the box being proportional to the weight of the study.

**Figure 3:**
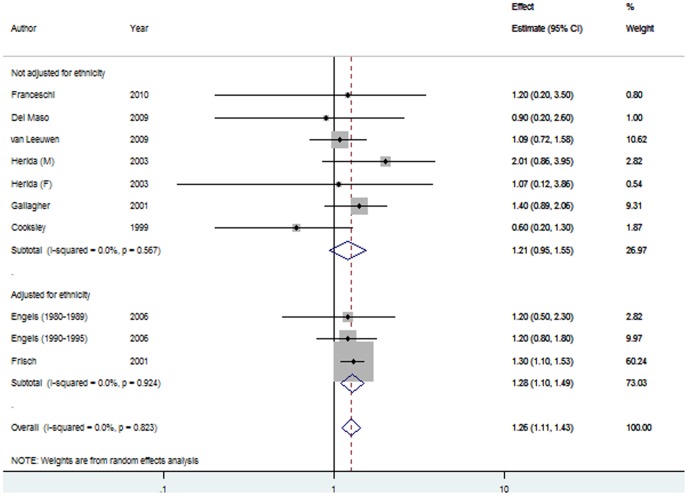
Forest plot of the association between HIV/AIDS and melanoma in the pre-HAART time period, stratified by adjustment for ethnicity. Each line represents an individual study result with the width of the horizontal line indicating 95% CI, the position of the box representing the point estimate, and the size of the box being proportional to the weight of the study.

The summary estimates for the association between HIV/AIDS in the post-HAART period was 1.26, 95% CI 0.97–1.64) and 1.50 (95% CI 1.12–2.01) when restricted to studies that accounted for ethnicity during analyses (Fig. 2), compared with a pre-HAART pRR of 1.26 (95% CI 1.11–1.43; P het = 0.82) and 1.28 (95% CI 1.10–1.49) for studies adjusted for ethnicity (Fig. 3). Significant heterogeneity was evident in the post-HAART era only (P = 0.004, I^2^ = 55.5; Table 2).

**Table 2:** Meta-analysis results using a Random Effects model: HIV/AIDS and risk of melanoma in the pre- and post-HAART time periods.

	Pre-HAART				Post-HAART			
	Number of studies	Pooled effect estimate (95% CI)	I2 (%)	P heterogeneity	Number of studies	Pooled effect estimate (95% CI)	I2 (%)	P heterogeneity
**HIV/AIDS Cohorts**	8	1.26 (1.11–1.43)	0	0.823	13	1.26 (0.97–1.64)	55.5	0·004
**Adjusted for ethnicity**								
Yes	2	1.28 (1.10–1.49)	0	0.924	6	1.50 (1.12–2.01)	54.5	0.051
No	6	1.21 (0.95–1.55)	0	0.567	7	1.03 (0.68–1.57)	46.6	0.051
**AIDS only Cohorts**	3	1.11 (0.95–1.30)	0	0.867	3	1.07 (0.83–1.39)	0	0.750
**Melanoma ascertainment**								
Internal/Registry	1	1.82 (0.90–3.65)	-	-	6	1.78 (1.17–2.71)	5.1	0.388
Registry only	7	1.25 (1.09–1.42)	0	0.821	7	1.11 (0.81–1.52)	67.8	0.002
**Population-based**								
Yes	8	1.26 (1.11–1.44)	0	0.823	10	1.15 (0.88–1.50)	56.4	0.007
No	0	-	-	-	3	2.98 (1.49–5.93)	0	0.759
**Study location**								
Europe	3	1.49 (0.85–2.61)	0	0.702	6	1.44 (0.98–2.11)	2.8	0.408
North America	4	1.27 (1.11–1.46)	0	0.584	6	1.50 (1.12–2.01)	54.5	0.051
Australia	1	1.09 (0.74–1.62)	-	-	1	0.61 (0.40–0.92)	-	-
**Study quality**								
High	6	1.26(1.11–1.44)	0	0.975	6	1.14 (1.83–1.57)	70.6	0.001
Low-moderate	2	1.14 (0.49–2.66)	48.4	0.144	7	1.61 (1.03–2.52)	16.9	0.297

For the pre-HAART time period, the summary estimates were not influenced by excluding one study at a time, with the pRR ranging from 1.21 (95% CI 0.98–1.48) with the omission of Frisch *et al.* [22] to 1.28 (95% CI, 1.12–1.47) with the omission of Van Leeuwen *et al.* [32]. The funnel plot was close to symmetrical and there was no evidence of publication bias using the Egger weighted regression method (P for bias = 0.38) or the Begg rank correlation method (P for bias = 0.72). Similarly, for the post-HAART time period, sensitivity analyses excluding individual studies resulted in summary estimates ranging from 1.20 (95% CI 0.90–1.59) with the omission of Silverberg *et al.* [38] to 1.38 (95% CI 1.10–1.74) with the omission of Van Leeuwen *et al.* [32]. Again, there was no evidence of publication bias (Egger P for bias = 0.58; Begg P for bias = 0.82).

For both time periods, the pRR was noticeably higher for studies that ascertained melanoma diagnosis using different methods for the HIV cohort and the comparison population (i.e. internal ascertainment for the HIV cohort and Registry ascertainment for the comparison population) (Table 2). In sub-group analyses restricted to studies that received a high quality score the summary estimate was higher in the pre-HAART period (pRR 1.26 pre-HAART and 1.14 post-HAART). In contrast, for studies that received a low-to-moderate quality score, the pooled estimate was higher in the post-HAART period (pRR 1.14 pre-HAART and 1.61 post-HAART).

Five studies did not present effect estimates stratified by time period [25], [26], [28]–[30], and the summary estimate including these five studies for all time periods (total n = 21) was 1·31 (95% CI, 1·11–1·55), with evidence of significant heterogeneity (P<0·001, I^2^ = 58%) (Figure S1). Also since several of the US studies used overlapping data, a sensitivity analysis excluding 2 of the 3 largest US studies in turn was conducted but showed little effect on the overall pooled estimate (for all time periods), ranging from 1.33 (with the exclusion of Simard et al. [36] and Engels et al. [27]) to 1.30 (with the exclusion of Simard et al. [36] and Fritsch et al. [22]).

The pRR for four studies that presented estimates of risk of melanoma in patients diagnosed with AIDS was 1.07 (95% CI 0.83–1.39), without significant heterogeneity (P = 0.75), and was similar in the pre-HAART and post-HAART time periods (Table 2). Removing the single study that had not adjusted for ethnicity [26] (contributed to the post-HAART estimate only) did not materially alter the pooled estimate (pRR 1.08, 95% CI 0.84–1.40).

## Discussion

We have systematically reviewed the most recent epidemiologic data reporting the relationship between HIV/AIDS and melanoma in the HAART era and conducted a meta-analysis. Taking into account the potential confounding effects of ethnicity, our findings show that risk of melanoma in those with HIV/AIDS remains elevated in the HAART era, with a 50% increased risk. We observed significant heterogeneity by study design, with a higher summary estimate from cohort studies where melanoma ascertainment differed between the HIV/AIDS cohort and the comparison population. Our findings for the pre-HAART period are consistent with those of a previous meta-analysis of 6 population-based studies [1] that was published in 2007. Our meta-analysis for the pre-HAART period included two additional studies [21], [24] and further follow-up data for three of the six study populations included in the previous review [31], [32], [34]. A second meta-analysis published in 2009 [42] was based on 10 studies that were also included in the current review. Thus the current study substantially extends the body of evidence reviewed as well as newly documenting the association in the HAART era.

A strength of our systematic review was the extensive quality assessment of relevant studies. Limitations included the potential for publication bias. Studies which were excluded from the meta-analysis because they did not provide essential data generally had a null result; therefore the meta-analysis may have over-estimated a more modest true association. On the other hand, the studies that were included may not have sufficiently covered the latent period for melanoma development (the time period between the onset of environmental exposure and tumor occurrence), leading to an underestimation of the association. Limited control of confounding in included studies may have influenced the results. For example, none of the included studies had data on key melanoma risk factors such as skin type, prevalence of melanocytic nevi, family history, though these factors are not associated with HIV/AIDS and are thus unlikely to have influenced the observed associations. Ethnicity, as a surrogate for skin colour, was accounted for in only 11 of the 21 included studies, yet was deemed essential for assessment of the true (unconfounded) magnitude of melanoma risk experienced by those with HIV/AIDS. Moreover, median follow-up time was not well reported by the included studies, and so was not able to be included in meaningful sensitivity analyses. We acknowledge the possibility that some cases in the post-HAART period may not have received the full recommended treatment as there were still controversies regarding treatment in the latter part of the 1990s [43]. There was also potential for double-counting of some US patient populations who contributed 51% of the weight to the pooled estimate in the post-HAART analyses, although it is very difficult to determine the existence or extent of overalap. A sensitivity analysis including in turn only one of the 3 largest US studies concerned, however, demonstrated little effect on the overall pooled estimate. Lastly, substantial heterogeneity was observed across studies. In subgroup analyses, although moderate heterogeneity still remained, the summary estimates showed positive relations in almost all subgroups. While melanoma incidence continued to rise amongst young men in nearly all parts of the world during the time period covered in these analyses, this would not have affected our results since included studies described melanoma risk in people with HIV/AIDS compared to the general population using standardized incidence ratios.

The increased risk of melanoma in populations with HIV/AIDS may be related to effects of HIV infection on the immune system although these are complex, including not only immunodeficiency, but also chronic immune activation and inflammation, and immune dysfunction and senescence [44]. We attempted to examine the influence of immunosuppression by examining the relationship in patients diagnosed with AIDS, characterized by severe immunodeficiency, however too few eligible studies were identified for the results to be informative. Similarly, there were insufficient data on duration of HIV infection, degree and duration of immunosuppression and duration of HAART to examine these factors in stratified analyses or meta-regression. One study presented results for risk of melanoma amongst individuals infected with HIV by recent CD4 count only, and reported elevated RRs for individuals with CD4 counts <200 and 201–499 cells/mL, but not for ≥500 cells/Ml [38]. Large-scale cohort studies with patient-level clinical data including CD4 count and use of HAART have the potential to better inform the management of melanoma risk (amongst other cancers) in populations with HIV/AIDS by better understanding the impact of immunosuppression, as well as how known risk factors operate differently in these populations versus the general population. Future collection of detailed clinical information on how melanomas are diagnosed (and whether it occurs as part of a physical examination for HIV/AIDS related symptoms), tumour location, and timing in relation to level of immunosuppression would greatly assist in understanding the relationship between the two diseases.

Immunosuppression is known to be associated with melanoma, shown by the increased risk of organ transplant recipients treated with immunosuppressive drugs [1]. Tumour-associated antigens are expressed by melanoma cells [45], and indirect evidence to support a role in immune surveillance derives from clinical observations including spontaneous tumour regression in some patients with primary melanoma [46] and regression associated with autoimmune skin depigmentation and vitiligo [47], observations that eventually led to the development of immunotherapeutic approaches for the treatment of metastatic disease [6], [48], [49]. Other possible explanations for the observed increased risk of melanoma include heightened medical surveillance for skin lesions including Kaposi's sarcoma, and high prevalence in the white HIV/AIDS population of lifestyle-related skin cancer risk factors such as high recreational sun exposure or use of tanning beds [5].

In summary, this meta-analysis points to a significantly increased risk of melanoma in HIV/AIDS populations in the HAART era. Whilst sub-group analyses revealed heterogeneity across some study characteristics, the pooled estimates in most sub-groups (most notable for studies that have appropriately adjusted for race/ethnicity and those rated of higher quality on the basis of our quality score) are indicative of a significantly increased risk. White skinned people with HIV/AIDS would benefit from regular screening of the skin for suspicious pigmented lesions, and since they also have a significantly increased risk of developing keratinocyte skin cancers (at least two-fold) [50] they should be counselled to avoid excessive sun exposure.

## Supporting Information

Figure S1 **Forest plot of the association between HIV/AIDS and melanoma (all studies).**
(TIF)

Table S1 **Meta-analysis results using the weighted average method: HIV/AIDS and risk of melanoma in the pre- and post-HAART time periods.**
(DOCX)

Table S2 **Assessment of the quality of the studies included in the meta-analysis of HIV/AIDS and risk of melanoma.**
(DOCX)

Appendix S1 **Search strategy to identify cohorts of patients with HIV/AIDS reporting on risk of melanoma.**
(DOCX)

Checklist S1 **PRISMA checklist.**
(DOC)
