# Supplementary figures and images for: Risk of Melanoma in People with HIV/AIDS in the Pre- and Post-HAART Eras: A Systematic Review and Meta-Analysis of Cohort Studies
Source: PLoS One. 2014 Apr 16;9(4):e95096. doi: 10.1371/journal.pone.0095096 (PMC3989294; doi:10.1371/journal.pone.0095096)

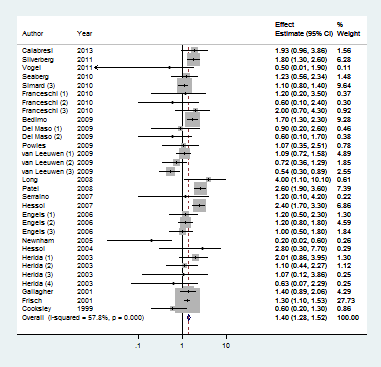

Supplement: Figure S1 — Forest plot of the association between HIV/AIDS and melanoma (all studies). (TIF) [file pone.0095096.s001.tif]
