# Supplementary material for: Risk of Melanoma in People with HIV/AIDS in the Pre- and Post-HAART Eras: A Systematic Review and Meta-Analysis of Cohort Studies
Source: PLoS One. 2014 Apr 16;9(4):e95096. doi: 10.1371/journal.pone.0095096 (PMC3989294; doi:10.1371/journal.pone.0095096)
Supplement: Table S1 — Meta-analysis results using the weighted average method: HIV/AIDS and risk of melanoma in the pre- and post-HAART time periods. (DOCX) [file pone.0095096.s002.docx]

**Table S1.** Meta-analysis results using the weighted average method: HIV/AIDS and risk of melanoma in the pre- and post-HAART time periods.

|  | **Pre-HAART** | | | |  | **Post-HAART** | | | |
| --- | --- | --- | --- | --- | --- | --- | --- | --- | --- |
|  | Number of studies | Pooled effect estimate (95% CI) | I^2^ (%) | P heterogeneity |  | Number of studies | Pooled effect estimate (95% CI) | I^2^ (%) | P heterogeneity |
|  |  |  |  |  |  |  |  |  |  |
| **HIV/AIDS Cohorts** | 8 | 1.26 (1.11-1.44) | 0 | 0.823 |  | 13 | 1.32 (1.14-1.52) | 55.5 | 0.004 |
|  |  |  |  |  |  |  |  |  |  |
| **Adjusted for ethnicity** |  |  |  |  |  |  |  |  |  |
| Yes | 2 | 1.28 (1.10-1.49) | 0 | 0.924 |  | 7 | 1.48 (1.26-1.74) | 51·6 | 0.054 |
| No | 6 | 1.21 (0.95-1.55) | 0 | 0.567 |  | 6 | 0.90 (0.67-1.20) | 36·5 | 0.126 |
|  |  |  |  |  |  |  |  |  |  |
| **AIDS only Cohorts** | 3 | 1.11 (0.95-1.30) | 0 | 0.867 |  | 3 | 1.07 (0.83-1.39) | 0 | 0.750 |
|  |  |  |  |  |  |  |  |  |  |
| **Melanoma ascertainment** |  |  |  |  |  |  |  |  |  |
| Internal/Registry | 1 | 1.82 (0.90-3.65) | - | - |  | 6 | 1.78 (1.19-2.67) | 5.1 | 0.388 |
| Registry only | 7 | 1.25 (1.09-1.42) | 0 | 0.821 |  | 7 | 1.26 (1.08-1.47) | 67.8 | 0.002 |
|  |  |  |  |  |  |  |  |  |  |
| **Population-based** |  |  |  |  |  |  |  |  |  |
| Yes | 8 | 1.26 (1.11-1.44) | 0 | 0.823 |  | 10 | 1.27 (1.10-1.47) | 56.4 | 0.007 |
| No | 0 | - | - | - |  | 3 | 2.98 (1.49-5.93) | 0 | 0.759 |
|  |  |  |  |  |  |  |  |  |  |
| **Study location** |  |  |  |  |  |  |  |  |  |
| Europe | 3 | 1.49 (0.85-2.61) | 0 | 0.702 |  | 6 | 1.44 (0.99-2.10) | 2.8 | 0.408 |
| North America | 4 | 1.27 (1.11-1.46) | 0 | 0.584 |  | 6 | 1.46 (1.24-1.72) | 54.5 | 0.051 |
| Australia | 1 | 1.09 (0.82-1.53) | - | - |  | 1 | 0.61 (0.40-0.92) | - | - |
|  |  |  |  |  |  |  |  |  |  |
| **Study quality** |  |  |  |  |  |  |  |  |  |
| High | 6 | 1.26 (1.11-1.44) | 0 | 0.975 |  | 6 | 1.27 (1.09-1.48) | 70.6 | 0.001 |
| Low-moderate | 2 | 1.22 (0.70-2.14) | 48.4 | 0.144 |  | 7 | 1.64 (1.11-2.42) | 16.9 | 0.297 |
|  |  |  |  |  |  |  |  |  |  |
|  |  |  |  |  |  |  |  |  |  |
