# Supplementary material for: Risk of Melanoma in People with HIV/AIDS in the Pre- and Post-HAART Eras: A Systematic Review and Meta-Analysis of Cohort Studies
Source: PLoS One. 2014 Apr 16;9(4):e95096. doi: 10.1371/journal.pone.0095096 (PMC3989294; doi:10.1371/journal.pone.0095096)
Supplement: Table S2 — Assessment of the quality of the studies included in the meta-analysis of HIV/AIDS and risk of melanoma. (DOCX) [file pone.0095096.s003.docx]

**Table S2:** Assessment of the quality of the studies included in the meta-analysis of HIV/AIDS and risk of melanoma.

| First author (published year) | Population-based | HIV diagnosis based on laboratory criteria | Calculation of person-years described | Melanoma ascertainment consistent in HIV cohort and comparison population | Adjusted for age, sex and ethnicity | Adjusted for other relevant factors | Total Score |
| --- | --- | --- | --- | --- | --- | --- | --- |
|  |  |  |  |  |  |  |  |
| Calabresi (2013) | 1 | 1 | 1 | 0 | 0 | 0 | 3 |
| Silverberg (2011) | 1 | 1 | 1 | 1 | 1 | 1 | 6 |
| Seaberg (2010) | 0 | 1 | 1 | 0 | 1* | 1 | 3 |
| Simard (2010) | 1 | 1 | 1 | 1 | 1 | 1 | 6 |
| Franceschi (2010) | 1 | 1 | 1 | 1 | 0 | 1 | 5 |
| Vogel (2010) | 1 | 1 | 0 | 0 | 0 | 0 | 2 |
| Bedimo (2009) | 0 | 1 | 1 | 1 | 1 | 1 | 5 |
| Del Maso (2009) | 1 | 1 | 1 | 1 | 0 | 0 | 4 |
| Powles (2009) | 0 | 1 | 1 | 0 | 0 | 0 | 2 |
| Van Leeuwen (2009) | 1 | 1 | 1 | 1 | 0 | 0 | 4 |
| Long (2008) | 0 | 1 | 0 | 0 | 1 | 1 | 3 |
| Patel (2008) | 1 | 1 | 1 | 0 | 1 | 0 | 4 |
| Serraino (2007) | 1 | 1 | 1 | 0 | 0 | 0 | 3 |
| Hessol (2007) | 1 | 1 | 1 | 1 | 1** | 1 | 5 |
| Engels (2006) | 1 | 1 | 0 | 1 | 1 | 1 | 5 |
| Newnham (2005) | 1 | 1 | 1 | 1 | 0 | 0 | 4 |
| Hessol (2004) | 0 | 1 | 1 | 0 | 1 | 0 | 3 |
| Herida (2003) | 1 | 1 | 0 | 0 | 0 | 0 | 2 |
| Gallagher (2001) | 1 | 1 | 1 | 1 | 1 | 1 | 6 |
| Frisch (2001) | 1 | 1 | 1 | 1 | 1 | 0 | 5 |
| Cooksley (1999) | 1 | 1 | 0 | 1 | 0 | 0 | 3 |
|  |  |  |  |  |  |  |  |

*Men only; ** Women only
