# Supplementary material for: Risk of Melanoma in People with HIV/AIDS in the Pre- and Post-HAART Eras: A Systematic Review and Meta-Analysis of Cohort Studies
Source: PLoS One. 2014 Apr 16;9(4):e95096. doi: 10.1371/journal.pone.0095096 (PMC3989294; doi:10.1371/journal.pone.0095096)
Supplement: Appendix S1 — Search strategy to identify cohorts of patients with HIV/AIDS reporting on risk of melanoma. (DOCX) [file pone.0095096.s004.docx]

**Appendix S1**. Search strategy to identify cohorts of patients with HIV/AIDS reporting on risk of melanoma.

PubMed

Medical subject headings terms

1. "Melanoma" or “Neoplasms”
2. “HIV” or "Acquired Immunodeficiency Syndrome"
3. “Cohort Studies” and “Etiology”
4. “Case Reports” or “Editorial” or “Letter” or “Comment” or “Therapeutics” or “Case-Control Studies”

Text terms

1. "cancer"
2. "AIDS” or “human immunodeficiency virus"
3. “cohort” or “risk”
4. “treatment”

Search String

1 AND 2 AND 3 NOT 4

Limits

Human Studies
